# Are There Advantages to Believing in Fate? The Belief in Negotiating With Fate When Faced With Constraints

**DOI:** 10.3389/fpsyg.2019.02354

**Published:** 2019-11-08

**Authors:** Evelyn W. M. Au, Krishna Savani

**Affiliations:** ^1^Department of Psychology, Singapore Management University, Singapore, Singapore; ^2^Culture Science Institute, Nanyang Business School, Nanyang Technological University, Singapore, Singapore

**Keywords:** negotiating with fate, agency, control, constraints, choice

## Abstract

Is cultural knowledge unique to a culture and inaccessible to other cultures, or is it a tool that can be recruited by individuals outside of that culture when the situation renders it relevant? As one test of this idea, we explored whether the applicability and benefits of a lay belief that originated from Chinese collective wisdom extends beyond cultural boundaries: *negotiating with fate*. Negotiating with fate postulates that fate imposes boundaries within which people can shape their outcomes through their actions. This belief contrasts fatalism, which has been traditionally interpreted as believing that fate dictates people’s life outcomes and renders their actions largely irrelevant. We found that the belief in negotiating with fate (but not fatalism) was strengthened when individuals recalled instances in which they were constrained, compared to when individuals recalled instances in which they were free to choose (Experiments 1 and 2). Subsequent studies found that after recalling a constraining event, exposure to the belief in negotiating with fate (but not exposure to fatalism) decreased repetitive thoughts (Experiment 3), increased the conviction that personal actions contributed to the event (Experiment 4), increased acceptance and positive reinterpretation of the event (Experiment 5), and increased how meaningful the event was (Experiment 6). Thus, when faced with constraints, acknowledging fate does not necessarily lead people to believe that their actions are irrelevant. Instead, when individuals face constraining circumstances in which potential courses of actions are clearly limited, they are more likely to believe that they are able to negotiate with fate, and this belief can help them move forward from negative outcomes. We found that the belief in negotiating with fate, although originating from Chinese folk culture, is spontaneously activated when people experience constraints even in a non-Chinese culture, and helps people cope with those constraints.

## Introduction

The Oxford dictionary defines a fated event as “*something that happens outside of a person’s control, regarded as pre-determined by a supernatural power*.” This conceptualization of fate, referred to as *fatalism*, assumes that fate has the ultimate authority over individuals’ lives and renders personal actions irrelevant. Past research revealed that Easterners believed in fate to a greater extent than Westerners (Cheng et al., 2013). This heightened belief in fate among Easterners would predict lower action and agency, but the strong academic achievement (Organisation for Economic Co-operation Development [OECD], 2018) and GDP growth (World Bank, 2018) in many Asian countries are somewhat inconsistent with this idea. To address this issue, recent research (Au et al., 2011, 2012, 2017) introduced an alternative perspective on fate: *negotiating with fate*. In contrast to fatalism’s assumption that people’s life outcomes are fixed and pre-determined, negotiating with fate postulates that fate imposes *boundaries* within which people can shape their outcomes through their actions. In other words, according to this belief, fate imposes constraints within which individuals must work (e.g., socio-cultural environments, socio-economic status, uncontrollable life events), but how individuals exercise agency within the boundaries of the constraints ultimately determines individuals’ life course. From this perspective, negotiating with fate prescribes specific roles for fate *and* personal control. In fact, we may routinely act consistently with the idea of negotiating with fate without realizing it. For example, when we open the fridge at the end of a long day to make dinner, we may decide to use the ingredients that we have available to make the best dinner possible, rather than to go to the supermarket and buy ingredients to make the specific dish that we are craving. Thus, in this situation, we are actively working with the constraints that we face (i.e., the ingredients that we have available) to achieve a desired goal (i.e., making a good dinner). The belief in *negotiating with fate* was identified by examining Chinese and American idioms (Au, 2008; Chiu et al., 2012). In both cultures, proverbs reflected three fate beliefs: (1) personal control (i.e., individuals’ actions solely determined outcomes and fate does not play a role; “You are the master of your fate”); (2) fatalism (i.e., fate determines outcomes, which cannot be altered by personal actions; “You will meet your fate on the road you took to avoid it”); and (3) negotiating with fate (i.e., fate imposes boundaries within which personal actions can shape outcomes; “When fate gives you lemons, make lemonade”). Here, we used an American idiom to illustrate the idea of negotiable fate, but a cross-cultural comparison revealed that Chinese proverbs are much more likely than American proverbs to capture the belief in negotiating with fate.

Given the origins of negotiating with fate, questions about its utility beyond the Chinese context may arise. Following Chiu and Hong’s (2005) approach to cultural knowledge, we argue for its pan-cultural applicability. Chiu and Hong (2005) likened cultural knowledge to tools: knowledge that helps the group adapt to the environmental challenges faced gains popularity; knowledge that is no longer perceived as useful in the current environmental context fades into obscurity. From this perspective, cultural knowledge is not unique to a culture and inaccessible to other cultures *per se*, but is a tool that can be recruited by individuals outside of that culture when the situation renders it relevant.

### Context-Specific Advantages of Negotiating With Fate

Au et al. (2011, 2012, 2017) proposed that the prevalence and consequences of different fate beliefs depended on the *socio-ecology* of the environment within which individuals function. More specifically, negotiating with fate was postulated as being particularly popular and beneficial when individuals must contend with external factors to determine outcomes. Au et al. (2012) examined the role of *constraints* on the consequences of negotiable fate, operationalizing constraints as acknowledging that there were aspects of the external world that cannot be changed through personal actions.

The cross-cultural results from Au et al. (2012) replicated past findings on perceived constraints (Su et al., 1999): Chinese participants perceived greater external constraints than their American counterparts. In Su et al.’s (1999) chapter, a study was reported in which Chinese participants subscribed to a stronger entity belief of the world (i.e., viewed the world as inherently static and unchangeable), but subscribed a stronger incremental theory of the self (i.e., viewed individuals to be more malleable), presumably because individuals need to change in response to the unchangeable external constraints. Furthermore, Chinese participants endorsed negotiating with fate to a greater extent than their American counterparts. Importantly, perceived constraints mediated the cultural difference in negotiating with fate, suggesting that constraints play a critical role in fostering the belief that individuals can change outcomes by working within boundaries imposed by fate. Lastly, the context-specific benefits of negotiating with fate were also evident: among those who perceived relatively greater constraints (i.e., the Chinese sample) negotiating with fate was positively associated with the greater use of active coping strategies and higher self-esteem; whereas among those who faced relatively fewer constraints (i.e., in the American sample), the same belief was associated with the greater use of *avoidant* coping strategies and lower self-esteem.

To test Chiu and Hong’s (2005) theory that cultural knowledge is flexibly recruited and applied in response to the environment, we examined whether the need to contend with constraints can cultivate the belief in negotiating with fate beyond the Chinese cultural context. If the results support their theory, we should be able to observe a similar pattern of results in a population that does *not* ordinarily believe that one can negotiate fate nor benefit from this belief.

### Strengthening the Endorsement of Negotiating With Fate and Its Associated Advantages

The current set of studies addressed two research questions. First, if the belief in negotiating with fate is part of a cognitive toolbox that individuals flexibly use in response to the situation, can the *experience of constraints* strengthen the belief that one can negotiate with fate, even among US American participants? Although Americans did not explicitly endorse the belief in fate (Leung et al., 2002, 2012), social psychologists have found that Americans were reluctant to tempt fate (Risen and Gilovich, 2008), and recruited fate as an explanation for past events when asked to think about “why” the event happened (Burrus and Roese, 2006). Therefore, we predicted that Americans’ belief in negotiating with fate will be stronger when they are faced with constraining circumstances (the *activation* hypothesis). We sought to capture the experience of facing daily constraints by operationalizing constraints as the lack of choices. *Constraints* and *lack of choices* have been found to be analogous, given research indicating that people experience the availability of choices as the lack of constraints (i.e., freedom) and the non-availability of choices as the presence of constraints (Schwartz et al., 2002; Savani et al., 2010). Thus, Studies 1 and 2 first activated a constraint (i.e., limitations on possible actions) vs. a choice (i.e., no limitations on possible actions) mindset (Savani and Rattan, 2012), and then assessed participants’ endorsement to negotiate with fate.

Second, among US American participants who do not generally believe strongly in negotiating with fate (Au et al., 2012), can exposure to the concept of negotiating with fate retain its advantages when they must contend with constraints? Au et al. (2012) found that the belief that one can negotiate with fate was not adaptive for Americans—those with stronger beliefs in negotiating with fate were more likely to use avoidant coping styles and had lower self-esteem. This is likely because unlike the Chinese, Americans face fewer constraints in their everyday lives. However, when the circumstances require Americans to navigate constraints, will the belief that one can negotiate with fate help them cope in a more positive manner? We expected that the answer to be yes (the *context-specific advantages* hypothesis). Thus, Studies 3–6 utilized a belief activation paradigm (adapted from Rattan et al., 2012) to explore whether activating the concept of negotiating with fate (vs. personal control or fatalism) affected various outcomes that indicate constructive ways of managing constraints: appreciating the impact of personal actions, ruminating less, viewing the event through more positive lenses, and finding meaning.

## Experiment 1

Experiment 1 tested the activation hypothesis by asking people to recall instances in which they made choices, in which they had to do something without any choice (i.e., constraints condition), or in which they just did something (a neutral condition with no reference to choice or the lack thereof). If we only included the two conditions in which participants recalled situations of choice vs. situations of constraints, we would be unable to establish whether changes in participants’ beliefs were driven by activating a choice mindset or by activating a constraints mindset. Given that our hypotheses specified the effects of activating a constraints mindset on negotiating with fate, we needed to include a neutral condition to test this effect. Participants then completed measures assessing the three types of beliefs identified by Chiu et al. (2012) analysis of Chinese and US American idioms: personal control, fatalism, and negotiating with fate. We predicted that recalling situations of constraint, rather than situations of choice, would strengthen people’s belief in negotiating with fate. We further expected that recalling situations of choice would strengthen people’s belief in personal control because the experience of recalling ways in which they were able to determine their own course of action should increase their belief that their actions determine personal outcomes. We did not expect that recalling situations of constraints would heighten people’s belief in fatalism compared to situations of choice. The rationale was that in situations of constraints, individuals are limited in what they could do, but their actions can still make a difference to their outcomes. Past research has found that experiencing lack of action-outcome contingency increases fatalism (Lefcourt, 1981, 1982), but we did not examine such extreme constraints. Under more everyday constraints (e.g., having to find an alternate way to get to work because one’s car broke down), individuals still had a chance to exercise personal control within the confines of the constraints.

### Method

#### Participants

A heterogeneous sample of 121 United States residents (representing different religions and levels of educational attainment; see Table 1 for demographics information) was recruited via Amazon’s Mechanical Turk.^1^ Participants who had taken part in any one experiment were excluded from participating in any subsequent experiments. Participants were randomly assigned to either the choice condition, the no-choice condition, or the neutral condition.

**TABLE 1:** Demographic information for all six samples.

	**Study 1**	**Study 2**	**Study 3**	**Study 4**	**Study 5**	**Study 6**
**Age**						
Mean	35.9	30.2	33.6	33.5	29.3	35.0
Standard deviation	11.9	8.9	10.8	11.8	8.7	12.9
Range	19–66	18–65	19–67	18–71	18–61	18–71
**Gender**						
Men	62	111	97	48	127	72
Women	59	89	120	90	67	86
**Educational attainment**						
Did not complete high school	1	1	1	2	1	2
Completed high school	18	18	26	14	18	14
Incomplete college degree	26	54	56	42	59	52
Associate’s degree	14	27	27	18	28	12
Bachelor’s degree	46	72	81	46	73	59
Master’s degree	12	21	22	15	11	16
Doctoral degree	4	5	4	2	3	2
Unreported	0	2	0	0	1	1
**Religious affiliation (*n*)**						
Non-religious	56	114	130	68	117	75
Protestant	25	27	21	22	25	27
Catholic	16	23	32	21	27	23
Christian	13	21	22	17	18	21
Other religions	11	15	12	10	6	12

*Frequencies are reported for gender, educational attainment and religious affiliation.*

#### Manipulation

To manipulate the salience of choice versus constraints, we adapted the manipulation used by Savani and Rattan (2012, Study 1). Participants completed a recall task about the four time periods: previous morning (8:00 a.m. to 12:00 p.m.), afternoon (12:00 p.m. to 4:00 p.m.), evening (4:00 p.m. to 8:00 p.m.), and night (8:00 p.m. to 12:00 a.m.). In the *neutral* condition, participants were asked to list five things they did during these four time periods; in the *choice* condition, participants were asked to list five choices they made (Savani and Rattan, 2012); and in the *no choice* condition, participants were asked to list five things that they did not have a choice but to do. The ease of recall might differ across conditions, and because the ease of recall might be associated with a greater sense of control, we asked participants the ease of completing the recall task, using a scale from 1 (*extremely difficult*) to 7 (*extremely easy*).

#### Dependent Measures

After the manipulation, participants completed three fate beliefs measures: personal control, fatalism, and negotiating with fate. To capture the belief in personal control (i.e., individuals’ actions solely determined outcomes and fate does not play a role), we used the internal locus of control measure (8 items; Levenson, 1981; sample item: “My life is determined by my own actions.”); to capture the belief in fatalism (i.e., fate dictates outcomes and personal actions do not have an impact), we used the chance locus of control measure (8 items; Levenson, 1981; sample item: “I have often found that what is going to happen will happen.”); to capture negotiating with fate, we used a revised the negotiating with fate measure that focuses more strongly on navigating boundaries imposed by fate (10 items; sample item: “I cannot change what fate has given me, but I can still achieve my dreams if I put in the effort.” See Appendix A). Participants indicated the extent of their agreement on a scale from 1 (*Strongly Disagree*) to 7 (*Strongly Agree*). Items from all three scales were completely randomized for each participant and presented on one page.

### Results

We averaged the items for each belief (8 items, α_PersonalControl_ = 0.79; 8 items, α_Fatalism_ = 0.84; 10 items, α_NegotiatingWithFate_ = 0.86). The means, standard deviations and 95% confidence intervals are presented in Table 2, and the correlations between the fate beliefs for each condition are presented in Table 3. We found significant differences between conditions in the ease of the recall task used in the manipulation, *F*(2,117) = 10.19, *p* < 0.01 (*M*_Neutral_ = 5.88; *M*_Choice_ = 5.15; *M*_NoChoice_ = 4.34, with higher numbers indicating greater ease), and thus, we included ease of recall as a covariate in all analyses.

**TABLE 2:** Means, standard deviations and 95% confidence intervals for the three lay beliefs about personal control and fate in Experiments 1 and 2.

	**Choice**				**Neutral**				**No choice**			
			**95% Confidence**				**95% Confidence**				**95% Confidence**	
	**Mean**	**S.D.**	**interval**		**Mean**	**S.D.**	**interval**		**Mean**	**S.D**	**interval**	
			**Lower**	**Upper**			**Lower**	**Upper**			**Lower**	**Upper**
**Study 1**												
Personal control	4.57	0.62	4.36	4.77	4.46	0.73	4.25	4.66	4.40	0.57	4.20	4.61
Fatalism	2.97	0.83	2.70	3.24	3.26	0.89	3.01	3.52	3.43	0.69	3.18	3.67
Negotiating with fate	4.47	0.61	4.28	4.67	4.41	0.64	4.23	4.60	4.72	0.45	4.56	4.88
**Study 2**												
Personal Control	4.00	0.55	3.86	4.13	4.00	0.58	3.86	4.14	4.14	0.54	4.01	4.28
Fatalism	2.89	0.67	2.72	3.06	2.98	0.64	2.83	3.14	2.88	0.75	2.69	3.06
Negotiating with Fate	4.90	0.54	4.77	5.03	4.90	0.53	4.77	5.03	5.05	0.55	4.92	5.19

**TABLE 3:** Correlations between fate beliefs, split by condition for Studies 1 and 2.

**Study 1**			**Study 2**		
		**Personal**			**Personal**
	**Fatalism**	**control**		**Fatalism**	**control**
**Choice**					
Negotiating with fate	–0.46^∗∗^	0.60^∗∗^	Negotiating with fate	−0.31^∗^	0.20
Fatalism	–	−0.49^∗^	Fatalism	–	–0.39^∗∗^
**Neutral**					
Negotiating with fate	–0.06	0.53^∗∗^	Negotiating with fate	–0.15	0.26^∗^
Fatalism	–	–0.56^∗∗^	Fatalism	–	–0.18
**No Choice**					
Negotiating with fate	0.12	0.43^∗∗^	Negotiating with fate	−0.27^∗^	0.44^∗∗^
Fatalism	–	−0.40^∗^	Fatalism		–0.33^∗∗^

*^∗∗^*p* < 0.01 and ^∗^*p* < 0.05.*

Our hypothesis was that the belief in negotiating with fate (but not belief in personal control or fatalism) would be higher in the no-choice condition compared with either the choice or neutral conditions. Given that we had specific hypotheses about how the dependent measures would vary across conditions, regressions testing those specific hypotheses have been recommended over conducting omnibus ANOVAs before conducting *post hoc* comparisons (Rosnow and Rosenthal, 1996; Abelson and Prentice, 1997). Thus, for each belief, we conducted one regression analysis to examine the effects of the no-choice constraints condition vis-à-vis the neutral and choice conditions. To this end, we entered two dummy variables identifying participants in the neutral and choice conditions, with the no-choice condition serving as the reference/comparison group. Note that a negative regression coefficient for either variable indicates that scores in that condition were lower than scores in the no-choice condition.

**FIGURE 1:**
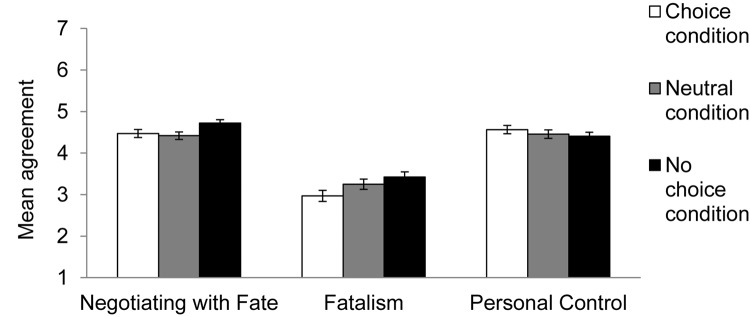
Mean agreement with lay theories about fate in Experiment 1, by condition. Error bars refer to standard errors of the means.

The belief in personal control did not differ between the neutral and no-choice conditions [*B* = −0.08, *SE* = 0.16, *t*(117) = 0.49, *p* = 0.63], or between the *choice* and no-choice conditions [*B* = 0.10, *SE* = 0.16; *t*(117) = 0.64, *p* = 0.52]. The belief in fatalism was weaker in the choice condition than in the no-choice condition [*B* = −0.40, *SE* = 0.19, *t*(117) = 1.98, *p* = 0.05, *d* = 0.60], but did not differ between the neutral and no-choice conditions [*B* = −0.049, *SE* = 0.20; *t*(117) = 0.25, *p* = 0.81]. Most importantly, the belief in negotiating with fate was stronger in the no-choice condition than in either the neutral [*B* = −0.34, *SE* = 0.14, *t*(117) = 2.53, *p* = 0.01, *d* = 0.58] or choice [*B* = −0.49, *SE* = 0.14, *t*(117) = 3.64, *p* < 0.001, *d* = 0.46] conditions (see Figure 1).

### Discussion

Experiment 1 supported our key hypothesis that, compared to situations in which people have freedom and choice, constraining situations (i.e., no choice) activated the belief that individuals can shape outcomes within the confines of the circumstances (i.e., negotiating with fate). Notably, constraining situations did not reduce people’s belief in personal control, indicating that even when they do not have choice, people were motivated to perceive that their actions determine outcomes. Predictably, constraining situations increased people’s belief in fatalism compared to the choice condition, but there were no differences in fatalism between the no choice condition and the neutral condition. In sum, Experiment 1 shows that the belief in negotiating with fate was spontaneously strengthened in situations of constraint.

## Experiment 2

Experiment 2 provided a conceptual replication of Experiment 1 while making the three control scales more comparable. The items for fatalism and personal control used in Experiment 1 measured beliefs about the role of fate (vs. personal control) for domain-specific outcomes (e.g., getting involved in a car accident) *and* non-domain specific outcomes (e.g., having control over one’s life); whereas, the items for the belief in negotiating with fate included primarily non-domain specific outcomes. This difference may make comparisons between scales potentially problematic. To remove this possible confound, we created a novel measure that simultaneously assessed all three constructs (i.e., personal control, fatalism and negotiating with fate) in parallel, using non-domain specific rather than domain-specific outcomes.

### Method

#### Participants

A sample of 200 United States. residents (see Table 1 for demographics information) was recruited via Amazon’s Mechanical Turk.^2^ Participants were randomly assigned to either the choice condition, the no-choice condition, or the neutral condition.

#### Procedure

Participants completed the same experimental manipulation as in Experiment 1, and then completed a novel measure with 7 sets of items (see Appendix B). Each set included a stem followed by three completing clauses that assessed fatalism, personal control and negotiating with fate. A sample set included the stem: “When individuals face a setback when pursuing a goal, they should believe that…” Participants were then asked to rate the extent to which they agree with each of the following: “…there’s only so much they can do to attain their goals” (*fatalism*), “…there’s nothing they cannot achieve” (*personal control*), and “…they can still attain their goals if they work with the current constraints” (*negotiating with fate*). Participants rated their agreement on a scale from 1 (*Strongly Disagree*) to 7 (*Strongly Agree*). The seven sets, and the three clauses representing each fate belief within each set, were presented in a random order to each participant.

### Results

We averaged the seven items for each belief (α_PersonalControl_ = 0.52; α_Fatalism_ = 0.73; α_NegotiatingWithFate_ = 0.81). The means, standard deviations and 95% confidence intervals are presented in Table 2, and the correlations between the fate beliefs for each condition are presented in Table 3. As in Experiment 1, there were significant differences between conditions in the ease of the recall task used in the manipulation, *F*(2,197) = 40.60, *p* < 0.01 (*M*_Neutral_ = 5.82; *M*_Choice_ = 4.75; *M*_NoChoice_ = 3.51), and thus, we included ease of recall as a covariate in all analyses. Two participants who did not answer the recall difficulty question were excluded from the following analysis, giving a total sample of 198.

**FIGURE 2:**
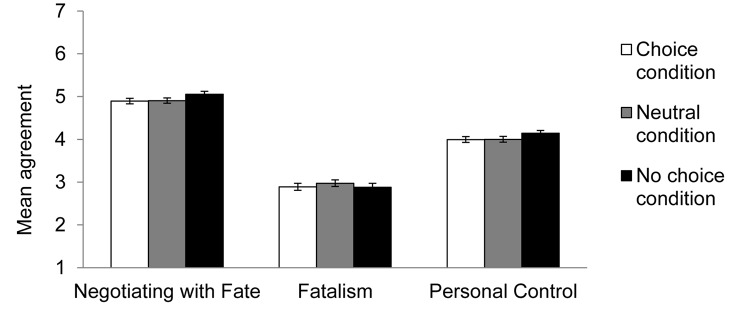
Mean agreement with lay theories about fate in Experiment 2, by condition. Error bars refer to standard errors of the means.

For each fate belief, we conducted a regression analysis to test whether that particular belief was different in the *choice* condition and the *neutral* condition compared to the *no choice* condition, which was treated as the reference group. Fatalism beliefs did not differ between the neutral and no-choice conditions [*B* = 0.15, *SE* = 0.14, *t*(194) = 1.07, *p* = 0.29], or between the choice and no-choice conditions [*B* = 0.04, *SE* = 0.13, *t*(194) = 0.29, *p* = 0.77]. Similarly, personal control beliefs did not differ between the neutral and no-choice conditions [*B* = −0.19, *SE* = 0.12, *t*(194) = −1.63, *p* = 0.10], or between the choice and no-choice conditions [*B* = −0.17, *SE* = 0.10, *t*(194) = −1.61, *p* = 0.11]. However, the belief in negotiating with fate was stronger in the no-choice condition (*M*_NoChoice_ = *5.10*) than in either the neutral [*M*_Routine_ = 4.86; *B* = −0.24, *SE* = 0.11, *t*(194) = 2.18, *p* = 0.05, *d* = 0.29] or choice [*M*_Choice_ = 4.90; *B* = −0.20, *SE* = 0.09, *t*(194) = 2.02, *p* = 0.03, *d* = 0.29] conditions (see Figure 2). This result replicates the finding from Experiment 1.

### Discussion

Using a more comparable measure of the three fate beliefs, Experiment 2 conceptually replicated the key finding of Experiment 1. Measuring the three fate beliefs at the same level of specificity and in parallel, Experiment 2 found that recalling in which they did not have choices (i.e., constraining situations) strengthened participants’ belief in negotiating with fate (but not their belief in personal control or fatalism). Taken together, findings from Experiments 1 and 2 provided support for the activation hypothesis: in constraining situations, individuals expressed a stronger endorsement of negotiating with fate.

## Experiments 3–6

Experiments 3 to 6 were designed to test whether the belief in negotiating with fate has context-specific advantages. That is, does activating the concept of negotiating with fate help people manage constraining situations? Thus, similar to the manipulation used in Experiments 1 and 2, participants were asked to recall a situation in their past that they had no choice but to face. We were interested in seeing how individuals respond in the present to past events for two reasons. First, individuals do “replay/relive” past life events over time to make sense of them (McAdams, 2005), and thus, how individuals come to see such events and how they cope with them evolve. Second, past research has shown how one’s current perspective on the past shapes their recollection of the past (Ross, 1989). Thus, we proposed that being able to change individuals’ present perspective on past events could help individuals cope with the event differently in the future.

To activate the beliefs in personal control, fatalism and negotiating with fate, we experimentally induced using a biased questionnaire method (Salancik, 1974). Although such beliefs are typically treated as individual differences, past research has shown that a number of beliefs simultaneously act as trait and state variables, and can be temporarily activated (Chiu et al., 1997; Rattan et al., 2012). Given the stronger causal claims that can be drawn from experiments, we manipulated whether a belief in personal control, fatalism, or negotiating with fate was salient.

To investigate the effects of the belief in negotiating with fate on people’s ability to move forward from a constraining event, we included a wide range of measures to evaluate the benefits of the belief. The goal of Experiment 3 was to test whether activating negotiating with fate would lead individuals to embrace its fatalistic component. Given that fatalism can hinder individuals’ ability to move forward from the event, Experiment 3 investigated the impact of a heightened belief in negotiating with fate on rumination. Rumination involves repeatedly mulling over the past event rather than thinking of how to avoid a similar occurrence in the future or how to manage the situation now that the event has occurred (Nolen-Hoeksema, 1991, 2000). In developing Experiments 4–6, we reasoned that even if we found that activating negotiating with fate decreased rumination, it is still unclear whether negotiating with fate fostered adaptive coping. More specifically, we cannot take the absence of rumination as implying the *presence* of coping strategies that would help individuals move forward from the event. Thus, Experiment 4 investigated negotiating with fate helped individuals identify their behaviors as having contributed to the event. If yes, this would suggest that individuals believed that they could potentially avoid a similar occurrence in the future by changing their behaviors rather than simply mulling over the event. These results would also imply that individuals did not view such events as inevitable, thereby providing additional support for the contention that negotiating with fate does not enhance people’s belief in fatalism.

Whereas Experiment 4 investigated people’s perceptions of how to potentially avoid a similar event in the future, Experiments 5 and 6 investigated how negotiating with fate helped individuals move forward now that the event has happened and cannot be changed. In Experiment 5, we examined whether negotiating with fate increases positive reinterpretation and acceptance of the event (but not denial or mental disengagement), and in Experiment 6, negotiating with fate increases a sense of meaning in the event after generating a silver lining, seeing the event as shaping who they are now.

By specifying the specific roles of fate and personal control, we expected negotiating with fate to provide context-specific benefits that are not afforded by personal control and fatalism. We reasoned that negotiating with fate highlights how individuals’ actions can still make a difference even when they face constraining circumstances that their actions cannot change. Thus, when individuals think of such situations, we predicted that negotiating with fate would decrease unproductive rumination (Experiment 3), and help them identify ways in which their actions contributed to an event (Experiment 4). Furthermore, by focusing on how to make the best of a constraining situation, we expected negotiating with fate to foster greater positive interpretation and acceptance of the event (but not denial and mental disengagement; Experiment 5). Lastly, we hypothesized that negotiating with fate helps people integrate the constraining event into their identities, after they search for a silver lining (Experiment 6).

## Experiment 3

The primary goal of Experiment 3 was to test whether exposure to negotiating with fate paralyzed individuals from moving forward after a constraining event. Rumination refers to self-focused and repetitive attention on one’s negative mood in response to the past event (Nolen-Hoeksema, 1991, 2000; Nolen-Hoeksema et al., 1999). Thus, rather than thinking about how they could potentially avoid a similar event in the future or how to manage the situation now that it has happened, individuals who ruminate repeatedly ask themselves questions such as, “What did I do to deserve this?” “Why can’t I handle things better?” “Why can’t I snap out of this?” Reported rumination levels were positively associated with stress levels and depressive symptoms after a multitude of experiences that individuals had no choice but to face (e.g., a natural disaster and after cancer surgery; Nolen-Hoeksema and Morrow, 1991; Yu et al., 2008). Given these findings, we investigated how activating negotiating with fate (vis-à-vis personal control and fatalism) would influence rumination levels.

We expected activating fatalism to increase rumination levels because past research has shown that perceiving an inability to direct important life events was associated with rumination (Nolen-Hoeksema et al., 2008). Based on this logic, activating the belief in personal control should lower rumination levels as it highlights the extent to which outcomes are determined by personal actions. However, the hypothesis was less straightforward: while some findings have shown that perceived control is associated with lowered rumination as suggested by the work of Nolen-Hoeksema and colleagues, others have found the reverse, specifically for events that the individual had no choice but to face (Meadows, 1989; Flett et al., 1991). Thus, contending with constraining situations may also be challenging for those exposed to the belief in personal control because such situations are at odds with the activated lay belief about fate. Given that studies on events that individuals had no choice but to face showed that personal control was associated with poorer reactions, we expected that activating personal control will also be associated with reports of greater rumination.

Importantly, we predicted that activating the belief in negotiating with fate will be associated with the lowest levels of rumination. Findings from previous research suggested that the belief in negotiating with fate provided individuals with a broader attributional framework for making sense of unexpected negative events, allowing them to register lower levels of surprise in response (Au et al., 2011; Study 1). Thus, we hypothesized that exposure to negotiate with fate will provide a similar context-specific benefit: help individuals move on from constraining events by lowering levels of rumination.

### Method

#### Participants

A sample of 217 United States residents (see Table 1 for demographics information) was recruited via Amazon’s Mechanical Turk.^3^

#### Event Recall

Similar to the no choice condition in Experiments 1 and 2, all participants were asked to recall an event from that they had no choice but to face, and to spend 5 min describing this event, and asked to spend 5 min describing this event. Participants recalled events such as experiencing financial difficulties, getting divorced, having health problems, and being involved in traffic accidents.

#### Manipulation

Upon completing the event recall task, we implemented the experimental manipulation using a biased questionnaire method (Salancik, 1974). Participants were randomly exposed to complete a scale that activated either the belief in personal control, fatalism, or negotiating with fate. The biased items were adapted from the measures used in Experiment 1 (see Appendix C). Examples of the biased questionnaires include, “For the most part, people’s lives are determined by their own actions” (α_PersonalControl_ = 0.83), “Occasionally, there are times that whatever is going to happen will happen – whether good or bad.” (α_Fatalism_ = 0.84), and “As long as people focus on making the best out of a bad situation, it doesn’t really matter what fate throws at you.” (α_NegotiatingWithFate_ = 0.92). The response scale accompanying the items was biased in that it included many more “agree” options than “disagree” options, thus encouraging participants to agree with the items presented: 1 = *do not agree*, 2 = *slightly agree*, 3 = *somewhat agree*, 4 = *moderately agree*, and 5 = *strongly agree* (Rattan et al., 2012).

#### Dependent Measure

To measure the extent to which participants ruminated about the stressful event that they recalled, we used the 22-item Ruminative Responses Style scale (Treynor et al., 2003). As the original scale captured individual differences in rumination, the instructions were adapted slightly to ask participants the extent to which they ruminated about the recalled event (sample item: “I think about “what did I do to deserve this”?”) Participants rated the frequency of their responses on a 4-point scale with 1 = *almost never*, 2 = *sometimes*, 3 = *often*, 4 = *almost always*.

### Results

In this study, we did not find any significant difference in mean agreement with the biased response scale across conditions [*M*_Fatalism_ = 3.64, *M*_PersonalControl_ = 3.42, *M*_NegotiatingWithFate_ = 3.55; *F(*2, 216) = 1.45, *p* = 0.23]. Yet, given that group differences in agreement were observed in the subsequent experiments, we included participants’ agreement to be biased scale as a covariate (as did Rattan et al., 2012, who used a similar paradigm) to address the concern that the results may be an artifact of greater agreement to certain beliefs rather than consequences of merely being exposed to different beliefs. To maintain consistency with Experiments 4–6, we controlled for participants’ agreement to the biased response scales in all analyses in this experiment as well.

**FIGURE 3:**
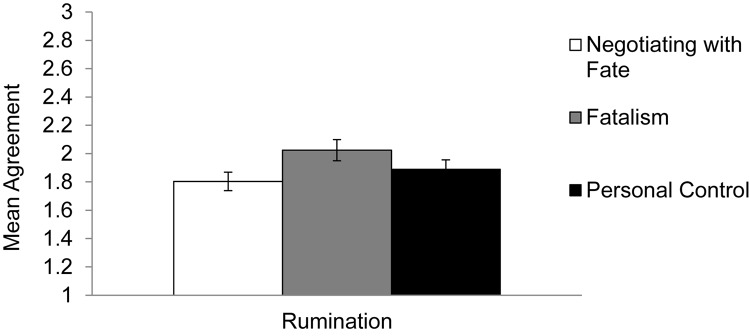
Mean rumination about constraining event in Experiment 4, by condition. Error bars refer to standard errors of the means.

We averaged the 22 items in the rumination scale (α = 0.93). The means, standard deviations, and 95% confidence intervals of the dependent variable are presented in Table 4. We then ran a regression testing whether rumination levels were different in the *fatalism* and *personal control* condition compared to the *negotiating with fate* condition, which was treated as the reference group. The results supported our hypothesis: activating negotiating with fate decreased rumination compared to activating fatalism [*B* = 0.22, *SE* = 0.09; *t*(213) = 2.25, *p* = 0.03, *d* = 0.38]. There was no significant difference between activating negotiating with fate and personal control [*B* = 0.08, *SE* = 0.09; *t*(213) = 0.87, *p* = 0.39, *d* = 0.15]. To compare the effects of activating personal control against fatalism, we excluded participants in the negotiating with fate group. Using the same regression equation, the results indicated that activating personal control did not lead to lower levels of rumination compared to activating fatalism [*B* = −0.13, *SE* = 0.10; *t*(143) = −1.31, *p* = 0.19, *d* = 0.23; see Figure 3].

**TABLE 4:** Means, standard deviations and 95% confidence intervals for Experiments 3–6.

	**Personal Control**				**Fatalism**				**Negotiating with Fate**			
			**95% Confidence**				**95% Confidence**				**95% Confidence**	
	**Mean**	**S.D.**	**interval**		**Mean**	**S.D.**	**interval**		**Mean**	**S.D**	**interval**	
			**Lower**	**Upper**			**Lower**	**Upper**			**Lower**	**Upper**
**Study 3**												
Rumination	1.89	0.59	1.75	2.02	2.02	0.61	1.88	2.17	1.80	0.56	1.67	1.93
**Study 4**												
Identifying own behavior as a contributing factor	3.33	1.12	3.03	3.68	3.43	0.97	3.14	3.72	3.87	1.17	3.52	4.22
**Study 5**												
Positive reappraisal	2.63	0.78	2.45	2.84	2.57	0.76	2.38	2.76	2.80	0.76	2.60	2.98
Acceptance	3.37	0.63	3.21	3.53	3.22	0.66	3.05	3.38	3.30	0.69	3.11	3.46
Denial	1.43	0.56	1.29	1.57	1.30	0.43	1.19	1.40	1.38	0.54	1.24	1.51
Behavioral disengagement	2.05	0.65	1.89	2.22	2.02	0.59	1.88	2.17	1.98	0.62	1.83	2.14
**Study 6**												
Meaning	4.09	1.42	3.71	4.47	4.04	1.48	3.61	4.46	4.55	1.60	4.10	4.99

### Discussion

Consistent with our hypotheses, Experiment 3 showed that activating the belief in negotiating with fate led to less rumination about a constraining event, compared to activating fatalism. The results also revealed that the rumination levels after activating personal control did not differ from *either* activating fatalism or negotiating with fate. Given the non-significant difference in reported rumination between the personal control and fatalism conditions, it appeared that using a set of explanatory factors that include *both* internal and external factors assisted individuals in reducing their repetitive thoughts about the constraining event more than the explanatory sets that involved either fatalism or personal control alone. Although lowered levels of rumination suggested that individuals were no longer dwelling on the event unconstructively, it would also be important to demonstrate that negotiating with fate also helped individuals actively *move forward* by assessing the extent to which they were able to accept and reinterpret the event in a more positive light.

## Experiment 4

The primary goal of Experiment 3 was to show that activating negotiating with fate did not lead individuals to embrace a fatalistic way of thinking, as evidenced by lowered rumination levels. However, it was still unclear whether negotiating with fate fosters adaptive coping. More specifically, we cannot take the absence of rumination as implying the *presence* of maintaining faith that their personal actions could make a difference in altering events. Thus, in Experiment 4, we explored the impact of negotiating with fate on identifying personal actions as contributing to the constraining event. If negotiating with fate led individuals to more readily rate their behaviors as contributing to the event, we would be able to draw two conclusions. First, by identifying their personal actions as contributing to the event, individuals also gained information about *how* they could potentially avoid a similar event in the future. This would show that negotiating with fate not only lowered individuals’ tendencies to mull repetitively over the event (Experiment 3), it also helped them move forward. Second, by identifying personal actions as a contributing factor to the event, individuals rejected the notion that the event was inevitable. Such a finding would provide further evidence that exposure to negotiating with fate did not enhance their beliefs in fate.

Past research has shown that believing that negotiating with fate helped individuals identify ways to make the use of available resources (Au et al., 2017), and thus, we argued that activating this belief would help individuals focus on aspects of a constraining situation that *can* be changed through actions (Au et al., 2012), such as their behaviors. Identifying ways in which personal actions contributed to the situation may provide basis for avoiding a similar occurrence in the future (Janoff-Bulman, 1979; Anderson and Riger, 1991), and rejects the notion that the outcome was inevitable (Roese, 1997). From this perspective, even if the cause was external, we proposed that believing that one can negotiate with fate would lead individuals to identify ways in which their actions could have potentially altered event’s occurrence (Mandel and Lehman, 1996).

In contrast, we expected neither the activation of the belief in personal control nor fatalism to increase perceptions that personal behaviors contributed to constraining events. Past research has shown that stronger beliefs in personal control were associated with a defensive reaction (i.e., thinking about how the outcome could have been worse; Kismatis and Wells, 2014), thus, reducing the role of behaviors in altering the situation. Similarly, a belief in fate strengthened perceived the inevitability of an event (Burrus and Roese, 2006), thus also rendering behaviors irrelevant. We proposed that negotiating with fate will help individuals focus on changeable aspects that contributed to the situation, rather than simply viewing *more* factors as contributing to constraining events. Thus, we explored an aspect of the individual that was viewed as relatively stable among North Americans (Su et al., 1999): one’s character. We expected that activating negotiable fate will increase ratings of behaviors (but not ratings of one’s character) as a contributing factor to the event.

### Methods

#### Participants

A sample of 139 United States residents (see Table 1 for demographics information) was recruited via Amazon’s Mechanical Turk.^4^

#### Event Recall

Participants were all asked to recall an event from their life that they had no choice but to face. To ensure that participants spend some time thinking about the event, we also asked them to think about ways they could have anticipated the course of the events and ways in which they could prevent a similar event from occurring in the future. Similar to Experiment 3, participants described events such as experiencing financial difficulties, relationship problems (e.g., infidelity and divorce), health problems, and car accidents.

#### Manipulation

Upon completing the recall task, we implemented the experimental manipulation using the same biased questionnaire method as in Experiment 3. Participants were randomly exposed to a scale that activated one of the three beliefs (α_Fatalism_ = 0.82; α_PersonalControl_ = 0.81; α_NegotiatingWithFate_ = 0.94).

#### Dependent Measure

We then asked participants to identify *why* the event happened, and the two types of potential factors were presented in a randomized order (Meyer and Taylor, 1986). The behavior scale consisted of four items, including, “I was too impulsive” and “I should have been more cautious” (α = 0.70).^5^ The *character* scale also consisted of four items, including, “I can’t take care of myself” and “I have bad luck” (α = 0.44). Participants were asked whether they agreed that each was a contributory factor on a 6-point scale (*1* = *strongly disagree*, *6* = *strongly agree*). Given the low internal reliability of the character subscale, we did not analyze the data for this dependent measure.

### Results

Consistent with Rattan et al. (2012), we tested if there is a difference in the mean agreement with the biased scales differed across conditions. We found a marginal difference in participants’ mean agreement with the biased scale across conditions [*M*_Fatalism_ = 3.54, *M*_PersonalControl_ = 3.67, *M*_NegotiatingWithFate_ = 3.90; *F*(2,136) = 2.89, *p* = 0.06]. Thus, we included participants’ agreement to be biased scale as a covariate (as did Rattan et al., 2012, who used a similar paradigm).

To compute the dependent measure, we averaged the four items in the behaviors (i.e., changeable factors) dependent variable. The means, standard deviations, and 95% confidence intervals of the dependent variable are presented in Table 4. We ran a regression to test whether the identification of behavior as a contributing factor differed between the *fatalism* condition and *personal control* condition compared to the *negotiating with fate* condition, which was treated as the reference group.

**FIGURE 4:**
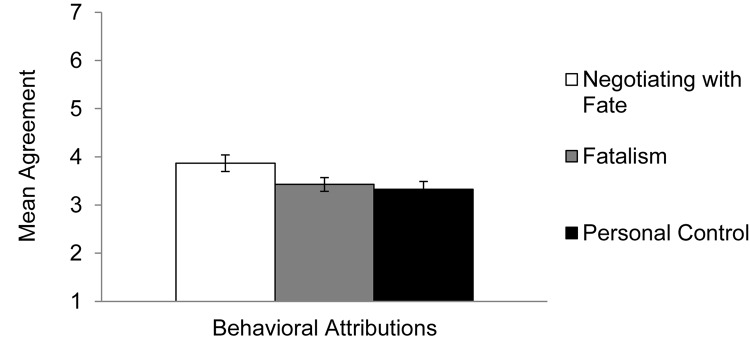
Mean agreement with behavioral attributions about constraining event in Experiment 4, by condition. Error bars refer to standard errors of the means.

The results supported our hypotheses (see Figure 4): activating negotiating with fate led participants to more readily identify behaviors as a contributing factor, compared to activating fatalism [*B* = −0.45, *SE* = 0.23; *t*(135) = 1.91, *p* = 0.06, *d* = 0.40], and activating personal control [*B* = −0.52, *SE* = 0.23; *t*(135) = 2.26, *p* = 0.03, *d* = 0.47]. Using the same procedure outlined for testing the effects of personal control vs. fatalism in Experiment 3, we conducted an additional regression. The findings indicated that activating fatalism did not lead participants to view behaviors as a contributing factor to a lesser extent than activating personal control [*B* = −0.05, *SE* = 0.22; *t*(90) = 1.31, *p* = 0.81, *d* = 0.10].

### Discussion

Experiment 4 tested the context-specific advantages of believing that one can negotiate with fate. The results showed that activating negotiating with fate helped people identify ways in which they could have potentially changed a recalled situation through their actions, compared to activating personal control or fatalism. These results supplemented the findings of Experiment 3 in two meaningful ways. First, these findings demonstrated that negotiating with fate helped individuals move forward from the event by identifying how changing their actions may help them avoid a similar event in the future. Thus, negotiating with fate lessened individuals’ tendencies to mull unproductively over the event (Experiment 3), and also identified *how* they could potentially prevent a similar event in the future. Second, these findings more directly tested our contention that negotiating with fate does not lead individuals to embrace its fatalistic component. Whereas Experiment 3 demonstrated that negotiating with fate lowered levels of rumination (which is commonly associated with fatalism), Experiment 4 revealed that negotiating with fate heightened the identification of personal actions in contributing to the event, thereby highlighting the importance of personal actions in altering the course of events while rejecting the notion that the event was inevitable.

The results from Experiments 3 and 4 suggested that despite the fatalistic component of the belief, believing in negotiating with fate (Au et al., 2012, 2017) and experimentally *activating* negotiating with fate had the same effect: it focused individuals’ attention on the changeable aspect of a constraining situation. Experiments 5 and 6 extended upon these findings by exploring how individuals may make the best of the event by through different coping strategies, including positive reinterpretation and acceptance (Experiment 5) and imbuing the event with meaning (Experiment 6).

## Experiment 5

As participants are recalling events from their past in our experiments, strategies involving attempts to actively change the circumstances surrounding that event may not be feasible. Thus, Experiment 5 focused on positive reappraisal and acceptance. In the past, these strategies were often examined in tandem: positive reappraisal referred to the individuals’ choice to perceive a constraining event in a more positive light, and acceptance referred to individuals’ ability to acknowledge that the event has occurred and cannot be changed. It is important to highlight the difference between acceptance and helplessness: whereas helplessness is characterized by passivity, acceptance requires the individual actively work toward being comfortable with the occurrence of the event (e.g., “I learn to live with it”). Thus, the active nature of these two coping strategies may explain why existing findings demonstrated that positive reappraisal and acceptance of negative situations leads to positive psychological outcomes (for a review, see Morling and Evered, 2006). For example, positive reappraisal of the situation helped parents better cope with the birth of high risk infants (Affleck et al., 1987), and acceptance of the situation was associated with more adaptive coping among pregnant women (Morling et al., 2003a, b). Therefore, these two can be considered to be *adaptive* coping strategies.^6^

We hypothesized that exposing individuals to personal control and fatalism would make it difficult for people to accept and positively reappraise constraining events, but for different reasons. The belief in personal control would propel individuals to find ways to *change* the existing situation rather than to accept the constraints as being unchangeable or finding ways to positively reinterpret the situation (Rothbaum et al., 1982; Morling et al., 2003a, b). The belief in fatalism was often associated with a sense of helplessness (Cheng et al., 2013), whereas acceptance and positive interpretation were strategies that individuals *actively* use to interact with constraints that they cannot change (Rothbaum et al., 1982). Given the link between fatalism and passive resignation, exposing participants to fatalism was unlikely to lead to greater acceptance and positive reinterpretation of the constraining event.

In contrast, we predicted that the belief in negotiating with fate would help individuals accept and positively reappraise a constraining event. Au et al.’s (2012) findings suggested that negotiating with fate helps individuals who face constraints by actively coping with the situation. However, the nature of “active coping” may differ between those who believe in negotiating with fate versus those who believe in personal control. As described above, those who believe in personal control are more likely to find ways to change the environment; in contrast, we expected those who believe in negotiating with fate to make the best out of the constraints that have been imposed by the circumstances. One such way was to accept that the circumstances cannot be changed, and to also actively search for ways to positively reappraise the situation.

To demonstrate that exposure to negotiating with fate increases acceptance and positive reinterpretation but not more traditionally passive strategies, we also included denial and mental disengagement (i.e., self-distraction) as dependent variables. If negotiating with fate heightensed the use of denial and mental disengagement *and* acceptance and positive reinterpretation, then may be negotiating with fate simply led to the greater usage of different strategies regardless of its level of passivity. Given that fatalism dictates that individuals cannot alter events through their actions, we expected that exposure to fatalism will increase the usage of denial and mental disengagement.

### Method

#### Participants

A sample of 194 United States residents (see Table 1 for demographics information) was recruited via Amazon’s Mechanical Turk.^7^ Participants were randomly assigned to either the fatalism, personal control, or negotiate with fate condition.

#### Event Recall

Using the same instructions as Experiment 4, participants were asked to recall an event in their past where they had no choice but to accept the situation.

#### Manipulation

Participants exposed to either the belief in fatalism (α = 0.86), personal control (α = 0.85), or negotiating with fate (α = 0.94) using the same biased questionnaire manipulated used in Experiments 3 and 4.

#### Dependent Measure

After the manipulation, we presented participants with four subscales from the COPE measure (Carver et al., 1989): positive reappraisal, acceptance, denial, and mental disengagement. The positive reappraisal scale measured the extent to which individuals could identify positive aspects of the constraining event (e.g., “I try to see it in a different light and make it more positive.”) The acceptance measure tapped the extent to which individuals acknowledged the occurrence of the constraining event (e.g., “I accept the reality that it has happened.”). The denial measure tapped individuals’ refusal to believe that the event had occurred (e.g., “I pretend that it never happened.”). Finally, the mental disengagement measure assessed the extent to which individuals used other aspects of their lives to distract themselves from the problem at hand rather than facing the situation (e.g., “I turn to work or other substitute activities to take my mind off things.”) Participants were asked to rate the extent to which they used these coping strategies in response to the recalled event, on a 4-point scale (1 = *Almost Never*, 2 = *Sometimes*, 3 = *Often*, 4 = *Almost Always*). Each subscale consisted of four items.

### Results

There was a significant difference in mean agreement with the biased scale across conditions [*M*_Fatalism_ = 3.58, *M*_PersonalControl_ = 3.40, *M_Negotia__t__e__With__Fate_* = 3.09; *F*(2,191) = 6.10, *p* = 0.003]. Therefore, we controlled for mean levels of agreement with the biased scale items used in the manipulation in the following analyses.

We averaged the four items of each subscale: positive reappraisal (α = 0.82), acceptance (α = 0.83), denial (α = 0.72), and mental disengagement (α = 0.65). The means, standard deviations, and 95% confidence intervals of the dependent measures are presented in Table 4. To test whether levels of positive reappraisal, acceptance, denial, and mental disengagement were different in the *fatalism* and *personal control* condition compared to the *negotiating with fate* condition (our reference group), we conducted a one regression for each dependent measure.

With regards to adaptive coping, participants reported significantly greater *positive reappraisal* of the event after negotiating with fate was activated compared to activating fatalism [*B* = −0.31, *SE* = 0.14, *t*(190) = 2.26, *p* = 0.03, *d* = 0.39], and marginally greater positive reappraisal when compared to activating personal control [*B* = −0.22, *SE* = 0.14, *t*(190) = 2.26, *p* = 0.10, *d* = 0.28]. For *acceptance*, participants reported marginally greater acceptance of the event after negotiating with fate was activated compared to activating fatalism [*B* = −0.22, *SE* = 0.11, *t*(190) = −1.81, *p* = 0.06, *d* = 0.10]. No differences in acceptance were found between the activating negotiating with fate and personal control [*B* = −0.02, *SE* = 0.11, *t*(190) = −0.20, *p* = 0.84].

With regards to passive strategies, for *denial*, no significant effects were found when we compared activating negotiating with fate to activating fatalism [*B* = −0.06, *SE* = 0.09, *t*(190) = −0.61, *p* = 0.54] or activating personal control [*B* = 0.05, *SE* = 0.09, *t*(190) = 0.63, *p* = 0.53]. Similarly, for *mental disengagement*, no significant effects were found when we compared activating negotiating with fate to activating fatalism [*B* = −0.00, *SE* = 0.11, *t*(190) = −0.08, *p* = 0.93] or activating personal control [*B* = 0.05, *SE* = 0.11, *t*(190) = 0.45, *p* = 0.65].

**FIGURE 5:**
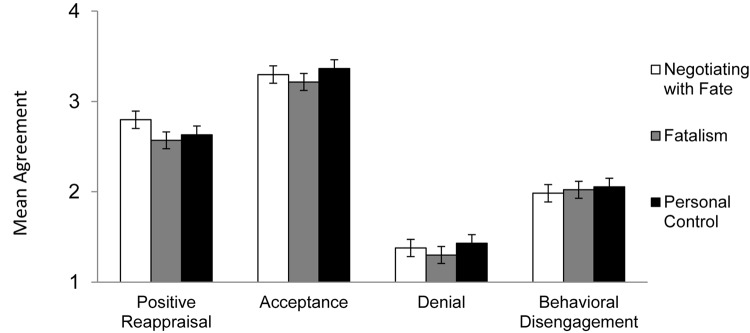
Mean positive reappraisal and acceptance of constraining event in Experiment 5, by condition. Error bars refer to standard errors of the means.

Using the same procedure outlined for testing the effects of personal control vs. fatalism in Experiments 3 and 4, we conducted additional regressions for each of the dependent variables. The findings indicated that activating personal control did not lead to any differences on the four dependent variables compared to activating fatalism {positive reappraisal [*B* = −0.09, *SE* = 0.14, *t*(127) = 0.62, *p* = 0.53]; acceptance [*B* = −0.20, *SE* = 0.11, *t*(127) = 1.79, *p* = 0.08]; denial [*B* = 0.13, *SE* = 0.09, *t*(127) = 1.45, *p* = 0.15]; or mental disengagement [*B* = 0.07, *SE* = 0.11, *t*(127) = 0.62, *p* = 0.54]; see Figure 5}.

### Discussion

Experiment 5 explored whether negotiating with fate helped people to move forward from the event through acceptance and positive reappraisal, two strategies that have been identified as adaptive when managing circumstances that are perceived as unchangeable. The results showed that activating this belief led participants to positively reinterpret the constraining event and accept that it has happened to a greater extent, when compared activating fatalism. To demonstrate that negotiating with fate does not simply increase the use of strategies regardless of passivity, our findings showed that activating negotiating with fate did not lead to the greater use of denial and mental disengagement. Surprisingly, exposure to fatalism did not increase the use of either denial or mental disengagement strategies. It may be possible that individuals who believe in fate use other passive strategies that we did not capture in the current studies.

In sum, given that there are no significant differences between activating fatalism and activating personal control, we proposed that acknowledging the need to make the best out of unchangeable constraints helped individuals find ways to see a constraining event more positively, and accepting it as part of one’s life course. Next, we explored one possible downstream consequence of positive reappraising the constraining event: finding meaning in the event by viewing it as an important part of their life story.

## Experiment 6

In Experiment 5, we found that activating negotiating with fate spontaneously led to higher levels of positive reappraisal. In Experiment 6, we investigated whether requiring individuals to actively search for a silver lining will eliminate the beneficial effects of negotiating with fate. If yes, it would suggest that the adaptive functions of negotiating with fate can be mimicked by introducing an intervention that simply requires individuals to reflect on positive consequences of the constraining event. Such findings would call into question the usefulness of the belief in negotiating with fate. In contrast, if we observed differences on the dependent measure, we would be able to conclude that negotiating with fate has advantages that extend beyond simply positively reappraising the event. The dependent measure in Experiment 6 explored one downstream consequence of positive reappraisals: the search for meaning. When individuals face situations that they have no choice but to face, they are likely to search for *meaning* behind this event. They are likely to ask: why did this event happen to *me* and what purpose does it serve? The search for meaning can be characterized as a process, which evolves as individuals are exposed to different perspectives on the intended consequence of the event for the individual. Generally, research has shown that the ability to identify silver linings of a stressful event allows people to integrate the event into their identity, and consequently, giving the experience a purpose (McAdams et al., 2001) and fostering adjustment (Davis et al., 1998). It is important to note that whereas positive reappraisal focuses solely on the positive consequences this event on their lives (e.g., “this event made me appreciate my loved ones more”), the search for meaning assess whether individuals integrated this event into their life history (e.g., “This event has shaped who I have become”), and identities (e.g., “this event shaped me into who I am today”).

The search for purpose generally helps individuals identify ways that the experience helped them become was an important part of their lives and defined who they are today. Thus, we reasoned that neither exposure to fatalism nor personal control will help individuals find greater meaning in the event, but for different reasons. From the fatalistic perspective, events occur simply because it is how fate has decreed it; these experiences do not serve any purpose for the individual. Therefore, it would be unlikely for a positive appraisal exercise to imbue greater meaning of a constraining event after activating fatalism. From the personal control perspective, events that befall an individual are solely determined by the self, and constraining events experiences threaten the potency of one’s ability to direct life events. This incongruence would make it difficult for individuals to incorporate these events into their identities, even after attempting to find a silver lining.

Importantly, we reasoned that engaging in positive interpretation of a constraining event would be most beneficial among those exposed to negotiating with fate. From this perspective, individuals acknowledge the need to make the best out of unchangeable constraining circumstances. Therefore, finding a silver lining allows individuals to identify lessons learned through dealing with these situations, making it easier for them to see how these events shaped them into who they are today.

### Method

#### Participants

A sample of 158 United States residents (see Table 1 for demographic information) was recruited via Amazon’s Mechanical Turk.^8^ Participants were randomly assigned either to the negotiating with fate, fatalism or personal control condition.

#### Measures and Procedures

Using the same instructions as previous experiments, participants were asked to recall an event in their past where they had no choice but to accept the situation. Participants were then randomly exposed to one of the biased questionnaires from Experiments 3–5 (α _Fatalism_ = *0.79*; α _PersonalControl_ = 0.83; α_NegotiatingWithFate_ = 0.92). Next, participants were further asked to reflect and think of a silver lining (i.e., something positive) that they can derive from the event. Participants also answered the following question, “Did thinking of a silver lining help you to see the negative event in a more positive light?” on a 9-point scale (1 = *it made it extremely difficult* to 9 = *it made it extremely easy*). This item was used as a covariate because we wanted to investigate whether the simple *act* of asking participants to search for a silver lining was sufficient to eliminate the beneficial effects of negotiating with fate. From a theoretical perspective, we wanted to test whether the mere completion of this activity was sufficient to mimic the effects of negotiating with fate, regardless of how helpful the participant found this exercise to be. From a statistical perspective, participants’ helpfulness ratings correlated significantly with meaning (*r* = 0.33, *p* < *0.001*), and thus, we needed to control for individual differences in the perceived helpfulness of the task. There were no significant differences in ratings of helpfulness across groups, *F*(3,154) = 0.944, *p* = 0.32.

#### Dependent Measure

Participants completed an 11-item scale about the meaningfulness of the event. The measure included two items of meaningfulness (“This event made me who I am today,” and “This event gave meaning to my life.”) from Kray et al. (2010), and additional items generated along these themes (e.g., “This event played a meaningful role in my life.”). Participants rated their agreement on a 7-point scale (1 = *Strongly Disagree* to 7 = *Strongly Agree*).

### Results

Although there were no mean differences in agreement with the control belief scale across conditions [*M*_Fatalism_ = 3.53, *M*_PersonalControl_ = 3.28, *M_Negotia__t__e__With__Fate_* = 3.28; *F*(2, 155) = 1.82, *p* = 0.16], mean levels of agreement to biased scale items were included as covariates to maintain consistency (as we did in Experiments 3–5). Given the positive correlation between the helpfulness rating and ascribed meaningfulness of the event, helpfulness ratings were also included in the regression analyses as a covariate, in the regressions reported below.

**FIGURE 6:**
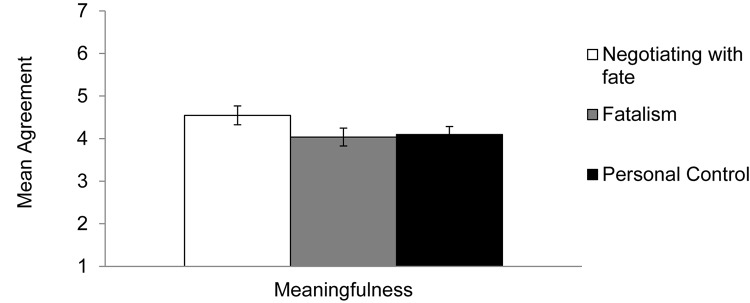
Mean meaningfulness of constraining event in Experiment 6, by condition. Error bars refer to standard errors of the means.

We first averaged the 11 items of the meaningfulness scale (α = 0.93). The means, standard deviations, and 95% confidence intervals of the dependent variable are presented in Table 4. We then tested whether levels of ascribed meaningfulness of the event were different in the *fatalism* condition and *personal control* condition compared to the *negotiating with fate* condition, which was treated as the reference group. These analyses revealed that participants ascribed greater meaning to the event after negotiating with fate was activated compared to activating fatalism [*B* = −0.56, *SE* = 0.28, *t*(153) = 1.97, *p* = 0.05, *d* = 0.38]. No significant difference in ascribed meaningfulness was found between activating negotiating with fate compared to activating personal control [*B* = −0.37, *SE* = 0.27, *t*(153) = 1.33, *p* = 0.19, *d* = 0.26]. Using the same procedure outlined for testing the effects of personal control vs. fatalism in Experiments 3–5, we conducted additional regressions for each of the dependent variables. The findings indicated that activating fatalism did not lead to different levels of ascribed meaning compared to activating personal control [*B* = 0.20, *SE* = 0.28, *t*(104) = 0.70, *p* = 0.49, *d* = 0.13; see Figure 6].

### Discussion

As hypothesized, Experiment 6 demonstrated that after attempting to positively reappraise a constraining event that individuals had no choice but to face, activating negotiating with fate (compared to activating fatalism) led people to ascribe greater meaning to the event. These findings indicated that acknowledging that one has to make the best out of unchangeable circumstances can help individuals incorporate experiences with constraints into their identities, making these experiences an important part of who they are today.

Furthermore, Experiment 5 demonstrated that negotiating with fate led to spontaneous positive reinterpretation of the constraining event. The results from Experiment 6 demonstrated even when individuals were required to search for a positive consequence of the constraining event, the unique advantages of positive reinterpretation after activating negotiating with fate cannot be reaped after activating fatalism. Interestingly, the effect of the silver lining task on the ascription of meaning for those exposed to personal control was midway between negotiating with fate and fatalism, and was not significantly different from either. This finding is consistent with those from Experiments 3 and 5 – whereas activating negotiating with fate consistently produces stronger advantages for coping, activating personal control did not lead to different levels of coping compared to negotiating with fate or fatalism. These results then lead us to two questions: (1) Is activating negotiable fate more beneficial than activating personal control for helping individuals move forward from the event? And (2) Is activating personal control more beneficial than activating fatalism for helping individuals move forward from the event? To this end, we turn to a meta-analysis conducted on our experiments.

## Meta-Analysis of the Effects Between Activating Negotiating With Fate and Personal Control

Experiments 3–6 demonstrated that, compared to activating fatalism, activating negotiating with fate helped individuals ruminate less (Experiment 3), identify changeable contributory factors to explain the constraining event (Experiment 4), use positive reappraisal and acceptance strategies (Experiment 5), and ascribe more meaning to the event (Experiment 6). However, does believing that one can negotiate with fate help people cope with constraining events significantly better than personal control? Activating negotiating with fate was seen as more beneficial than activating personal control only in Experiment 4, in which those exposed to negotiating with fate were more likely to identify their behaviors as having contributed to the event. Activating personal control did not differ from activating negotiating with fate in the other experiments. Thus, to address this question, we conducted a meta-analysis on the results of Experiments 3–6. To this end, we used the Exploratory Software for Confidence Intervals [“ESCI for Macintosh (Excel^©^ 2011)” worksheet]^9^ for computing meta-analyses accompanying Cumming (2012). For each study, we first saved the residuals after controlling for any covariates. We included the primary dependent measure from each experiment: rumination (Experiment 3), behavioral attributions (Experiment 4), positive reappraisal (Experiment 5), and sense of meaning (Experiment 6), with all variables were recoded such that higher numbers indicate a more adaptive response to the constraining event. We ran a random-effects meta-analysis model assuming that the effect size of the difference between conditions are likely to vary across the different dependent measures.

When comparing activating negotiating with fate against activating fatalism, the estimated meta-analytic effect size of the difference between the two (weighted by sample size) was significant, with estimated effect size of difference between group means (see Cumming, 2012) *ES* = 0.2844, *z* = 3.949, *p* = 0.0001, *var(ES)* = 0.0052, 95% CI[0.1432, 0.4255]. The estimated heterogeneity in effect sizes across studies was non-significant, *Q*(*df* = 3) = 1.6616, *p* = 0.65. When comparing activating negotiating with fate against activating personal control, the mean meta-analytic effect size of difference between the two was also significant, *ES* = 0.2022, *z* = 2.346, *p* = 0.019, *var(ES)* = 0.0074, 95% CI[0.0333, 0.3712]. The estimated heterogeneity in effect sizes across studies was non-significant, *Q*(*df* = 3) = 3.8017, *p* = 0.28.

In contrast, the mean meta-analytic effect size of difference between fatalism and personal control was non-significant, *ES* = −0.0977, *z* = 1.344, *p* = 0.179, *var(ES)* = 0.00528, 95% CI[−0.2401, 0.0448]. The estimated heterogeneity in effect sizes across studies was again non-significant, *Q*(*df* = 3) = 1.1754, *p* = 0.76.

### Discussion

Importantly, the meta-analyses showed that activating the belief in negotiating with fate provided more context-specific advantages for moving forward from a constraining event, compared to either the activation of fatalism or personal control. Given its description, negotiating with fate could be mistaken as an intermediate construct that represents the “middle ground” on a continuum, with believing in fatalism on one end and personal control on the other. A “middle ground” construct of this nature could capture two disparate beliefs: the *lack* of belief in either (i.e., disbelief in either fatalism or personal control) or a belief in *both* (i.e., believing in *both* fatalism and personal to some extent). Regardless of its constitution, one would expect a middle ground construct to produce effects that fall consistently between those of fatalism and personal control.

Instead, the meta-analytic findings suggest that negotiating with fate was associated with a divergent pattern of results when compared to fatalism and personal control, whereas activating fatalism and personal control do not significantly differ from each other in terms of their effects on responses to the recalled constraining situations. These findings provide support for the way we conceptualize negotiating with fate as prescribing *specific* roles for fate and personal control, a belief that cannot be adequately captured as simply a *combination* of fatalism and personal control.

## General Discussion

The belief in negotiating with fate has been found to be popular and beneficial in the Chinese cultural context (Au et al., 2011, 2012, 2017). The present series of six experiments explored the context-specific applicability and advantages of the belief in negotiating with fate beyond the Chinese cultural context. Our results provided support for Chiu and Hong’s (2005) theory that the advantages of cultural knowledge can extend beyond its originators, and that the recruitment and usage of such knowledge is dependent on the *situational* experiences of the individual. Consistent with our hypotheses, we found that: (1) the experiences of constraints activate a belief that one can negotiate with fate, which refers to acknowledging that external factors impose a boundary within which individuals can use their personal actions to shape outcomes (the *activation* hypothesis); and (2) when individuals face constraints in their lives, this belief is beneficial for helping them move forward from manage constraining life events that they had no choice but to fate (the *context-specific advantages* hypothesis). The meta-analysis of Experiments 3–6 indicated that overall, negotiating with fate provides greater context-specific advantages than fatalism or personal control alone.

### Theoretical Implications

#### Implications for Studying Cultural Knowledge as a Universal Cognitive Toolbox

Cheng et al. (2013) conducted an extensive literature review on the detrimental effects of believing that external factors determine personal outcomes. The overarching conclusion was that such beliefs have a less detrimental effect on mental health among Easterners (e.g., Chinese) compared to Westerners (e.g., US Americans). The authors cited negotiating with fate as a hybrid belief in fate and personal control that could potentially explain this cultural difference. Indeed, Au et al. (2012) showed that negotiating with fate was stronger among the Chinese participants and associated with markers of positive (rather than negative) psychological functioning.

Extending these findings, we proposed that the belief that one can negotiate with fate can be similarly popular and beneficial among individuals from the West. Although negotiating with fate originated from collective Chinese wisdom, we followed Chiu and Hong’s (2005) approach to cultural knowledge and we argue for its universal applicability. Triangulating environmental and historical factors illuminated why different cultural practices evolved, and the purpose served by different cultural practices and beliefs (Gelfand et al., 2011). Building on past work identifying perceived constraints as the mediator for the cultural difference in the belief that one can negotiate with fate (Au et al., 2012), the current research investigated its causal impact when people are faced with constraints. The results from our current studies provided support for the argument that cultural beliefs have universal applicability when factors engendering these beliefs are salient in other cultures. Thus, even among individuals who were not embedded within the culture from which a belief originated, this belief can still be flexibly recruited and used to individuals’ advantage.

#### Implications for Beliefs in Fate

The mere mention of “believing in fate” may conjure up images of individuals who have lost all hope for shaping their lives. Indeed, past research tended to view fatalistic beliefs, defined as viewing fate as the sole determinant of outcomes, as largely irrational and maladaptive (Mayo et al., 2001; Dettenborn et al., 2004). However, the construct of *negotiating with fate* suggested that acknowledging fate’s role in determining outcomes does not necessarily render personal actions irrelevant. Thus far, research on negotiating with fate identified two ways in which individuals’ actions can still make a difference despite the impact of external factors. First, the current studies explored the impact of constraints, defined as working with the boundaries that are imposed by external factors. In the context of constraints, individuals’ personal actions still matter despite the impact of external factors. Specifically, individuals’ outcomes depended on whether they are able to make the best out of the situation they are given. Second, Au et al.’s (2017) studies explored the context of uncertainty, defined as the unpredictability of a positive outcome. For example, winning the lottery is unpredictable because individuals do not know the winning numbers ahead of purchasing one. Therefore, whether individuals’ actions (e.g., buying a lottery ticket) will lead to a positive outcome is unknown, but the individual’s non-action (e.g., *not* buying a lottery ticket) will guarantee failure. In this context, an individual’s actions are important for preventing failure, even if these actions do not guarantee success. Distinguishing between qualitatively different experiences with external factors has important implications for advancing our understanding of how individuals remain resilient. We proposed that sustained interaction with unyielding external factors that leave no room for personal actions to alter one’s life course would engender a belief in fatalism. A nuanced examination of divergent effects of external factors will help the field refine its theories and hypotheses regarding the beliefs that foster adaptive coping under a wider range of conditions.

### Limitations and Future Directions

#### Investigating How Negotiating With Fate Increases Positive Appraisals

In Experiment 5, the results suggested that activating negotiating with fate increased levels of positive appraisal. However, surprisingly, those exposed to negotiable fate in Experiment 6 did not rate the generated silver lining as being more helpful than those exposed to fatalism or personal control. Future studies may wish to unpack the nature of this effect. For example, the instructions for Experiment 6 only asked participants to generate one silver lining and rate the helpfulness of that particular silver lining. This leaves open the possibility that those exposed to negotiating with fate were better able to positively reappraise the event (in Experiment 5) because they spontaneously generated more than one silver lining, and not because they found just one silver lining *more* helpful. Perhaps future studies can require participants to generate as many silver linings as possible, and explore whether the number of silver linings mediates the relationship between activating negotiating with fate and positive reappraisal or meaningfulness of the event.

#### Investigating How Activating Different Beliefs Shape Other Coping Strategies

In Experiment 5, we asked participants the frequency with which they used four types of coping strategies. Future research may wish to simply ask individuals who they tended to cope with the past event in an open-ended manner, and thus, be able to capture differences in coping strategies beyond the ones that we have already included.

#### Generalizability Across Situations of Constraints

In our experiments, we asked participants to recall an event in their lives in which they had no choice but to face a particular situation, thus focusing on *specific situations* of constraints. Future studies can consider exploring other operationalizations of constraints, including socio-economic status (Snibbe and Markus, 2005), lack of residential mobility (e.g., Oishi, 2010; Oishi et al., 2012; Oishi et al., 2007a, b), and readiness of one’s society to punish deviance (i.e., tightness vs. looseness; Gelfand et al., 2011). These future avenues of research also offer a chance to unpack the overlap between negotiating with fate and personal control. Perhaps in managing unchanging structural and institutional constraints, both beliefs would lead individuals to active (rather than passive) in goal attainment attempts. However, those who believe in negotiable fate may be more likely to choose and find strategies that allow them to attain their goals without upending the constraints – a hypothesis consistent with acknowledging that fate has imposed unchangeable boundaries, but individuals can do the best that they can within these limitations to achieve desired outcomes. In contrast, those who believe in personal control may be more likely to attain goals by instigating changes that will *remove* the constraints to bring about desired outcomes. Thus, a more nuanced investigation into how goal pursuit in the face of constraints may shed greater light onto the differences between negotiating with fate and personal control.

### Conclusion

Divergent cultural landscapes foster collective wisdom that enables individuals to survive and thrive in that particular context. Although fate tends to be less prevalent in the West, our research showed that US Americans flexibly recruited a belief rooted in Chinese collective wisdom –negotiating with fate– to help them navigate constraints. Thus, when external factors loom large, individuals have a tendency to work *with* these factors rather than surrender to them. By identifying factors that shape fate beliefs, we highlighted how cultural beliefs can have pan-cultural implications.

## Data Availability Statement

The raw data supporting the conclusions of this manuscript will be made available by the authors, without undue reservation, to any qualified researcher.

## Ethics Statement

The protocols for all reported studies were approved by the Institutional Review Board at Singapore Management University, National University of Singapore, or Nanyang Technological University, and the studies were carried out in accordance with the policies of the university institutional review boards and national guidelines. All participants were given written consent in accordance with the Declaration of Helsinki.

## Author Contributions

EA analyzed the data and drafted the manuscript. KS made substantial edits. Both authors designed the experimental studies, collected the data, and approved the final version of the manuscript.

## Conflict of Interest

The authors declare that the research was conducted in the absence of any commercial or financial relationships that could be construed as a potential conflict of interest.

## Footnotes

1 According to Gervais et al. (2015), the average effect size in psychology is *d* = 0.40. With this average size, *N* = 100 is required to achieve 80% power with alpha = 0.05. Thus, the target sample size for Experiment 1 was 100 participants per cell. A survey seeking 300 United States residents was posted on www.mturk.com, and 208 participants completed the study by the time the study expired. In all six experiments, we identified non-attentive participants with the same three checks. First, we used an instructions check before random assignment to identify participants who tend not to read instructions (Oppenheimer et al., 2009). Second, at the end of the experiment, we asked participants if they were eating or drinking during the survey (binary forced-choice question) and thus were likely to be distracted. Third, we administered a multiple-choice memory test about the task that they completed in the manipulation, again to identify participants who were not paying attention during the survey. Forty-four participants were excluded for failing the instructions check, 27 participants were excluded for failing the distractors check, and 16 were excluded for failing the memory check. These criteria and extent of exclusions are typical for online samples (e.g., Hardisty and Weber, 2009). Conducting research online offers the advantage of much more diverse samples but has the disadvantage of the lack of control over the amount of distraction experienced by participants during the survey. Excluding data from the inattentive participants increases statistical power (e.g., Maniaci and Rogge, 2014; Oppenheimer et al., 2009) without changing the direction of the underlying effects.

2 The target sample size for Experiment 2 was also 100 participants per cell. A survey seeking 300 United States residents was posted on www.mturk.com, and 302 participants completed the study before it expired. Non-attentive participants were identified using the same criteria described in Experiment 1. Fifty-five participants were excluded for failing the instructions check, 37 participants were excluded for eating or drinking, and 10 were excluded for failing the memory check.

3 The target sample size for Experiment 3 was also 100 participants per cell. A survey seeking 300 United States residents was posted on www.mturk.com, and 302 participants completed the study before it expired. Non-attentive participants were identified using the same criteria described in the previous three experiments. Fifty-four participants were excluded for failing the instructions check, 27 participants were excluded for eating or drinking, and 3 were excluded for failing the memory check. One participant was excluded for not completing the experimental manipulation. Due to a programming error, participants were not asked when the event occurred, and thus we could not exclude participants who recalled an event more than a decade old.

4 The target sample size for Experiment 4 was also 100 participants per cell. A survey seeking 300 United States residents was posted on www.mturk.com, and 308 participants completed the study before it expired. Non-attentive participants were identified using the same criteria described in Experiments 1 and 2. Sixty-two participants were excluded for failing the instructions check, 29 participants were excluded for eating or drinking, and 54 were excluded for failing the memory check. As past research has demonstrated individuals’ coping strategies in response to a negative situation remains relatively stable between the event and up to a decade (Pollard and Kennedy, 2007), only participants who listed an event that occurred within the last 10 years was included in the sample and analyses. Twenty-four participants were excluded from the sample as the event they recalled occurred more than 10 years ago, and one participant did not report the year when the event occurred.

5 Meyer and Taylor (1986) reported a 5-item scale, which consists of the 4-item scale used in our study and one additional item (i.e., “I am too trusting”). Meyer and Taylor (1986) designed the scale to specifically assess attributions in response to being raped, but this particular behavior is too specific and unlikely to be applicable in the wide range of constraining events that were recalled by our participants (e.g., car accident, health problem, financial difficulties, divorce). Therefore, this item was not included in the dependent measure in our study.

6 Generally, research has shown that acceptance is an adaptive coping strategy (for a review, see Morling and Evered, 2006), but some research has demonstrated that acceptance is associated with depressive symptoms (e.g., Garnefski and Kraaij, 2006). Morling and Evered (2006) distinguished between two different kinds of acceptance: acceptance by highlighting the hopelessness of the situation (which is maladaptive) or adjusting the self to the unchangeable reality (which promotes psychological adjustment). Garnefski and Kraaij’s (2006) measured acceptance in terms of resignation, and therefore, the positive association between acceptance and depressive symptoms is unsurprising. Instead, we measure acceptance in terms of acknowledging the occurrence of the event rather than resignation, which is associated with positive coping outcomes (Carver et al., 1989).

7 The target sample size for Experiment 5 was also 100 participants per cell. A survey seeking 300 United States residents was posted on www.mturk.com, and 299 participants completed the study before it expired. Non-attentive participants were identified using the same criteria described in the previous four experiments. Fifty-eight participants were excluded for failing the instructions check, 28 participants were excluded for eating or drinking, 3 were excluded for failing the memory check. Consistent with Experiments 3 and 15 were excluded for listing an event that occurred more than 10 years ago. One additional participant was excluded for not completing the experimental manipulation.

8 The target sample size for Experiment 6 was also 100 participants per cell. A survey seeking 300 United States residents was posted on www.mturk.com, and 299 participants completed the study before it expired. Non-attentive participants were identified using the same criteria described in the previous five experiments. Eighty-six participants were excluded for failing the instructions check, 24 participants were excluded for eating or drinking, and 5 were excluded for failing the memory check. Consistent with Experiments 3, 5, and 26 were excluded for listing an event that occurred more than 10 years ago.

9 Retrieved from http://www.latrobe.edu.au/psy/research/cognitive-and-developmental-psychology/esci, version dated July 4, 2011, downloaded on 8 February, 2014.
