# Corrigendum: Are There Advantages to Believing in Fate? The Belief in Negotiating With Fate When Faced With Constraints

**DOI:** 10.3389/fpsyg.2019.02949

**Published:** 2020-02-24

**Authors:** Evelyn W. M. Au, Krishna Savani

**Affiliations:** ^1^Department of Psychology, Singapore Management University, Singapore, Singapore; ^2^Culture Science Institute, Nanyang Business School, Nanyang Technological University, Singapore, Singapore

**Keywords:** negotiating with fate, agency, control, constraints, choice

In the original article, there was an error. In Experiment 1, there was an error in reporting the mean for the ease of the recall task in the Neutral condition. It should have been “*M*_Neutral_ = 5.88”, instead of “*M*_Neutral_ = 5.19”, as originally reported.

A correction has been made to Experiment 1, Results section, paragraph one:

“We averaged the items for each belief (8 items, α_PersonalControl_ = 0.79; 8 items, α_Fatalism_ = 0.84; 10 items, α_NegotiatingWithFate_ = 0.86). The means, standard deviations and 95% confidence intervals are presented in **Table 2**, and the correlations between the fate beliefs for each condition are presented in Table 3. We found significant differences between conditions in the ease of the recall task used in the manipulation, *F*(2,117) = 10.19, *p* < 0.01 (*M*_Neutral_ = 5.88; *M*_Choice_ = 5.15; *M*_NoChoice_ = 4.34, with higher numbers indicating greater ease), and thus, we included ease of recall as a covariate in all analyses.”

In **Experiment 2**, there was an error in reporting the *p*-value when comparing the strength of belief in negotiating with fate across the no choice condition and the routine and choice conditions (incorrectly reported as *p* = 0.05 and *p* = 0.03, respectively). The *p*-values should have been *p* = 0.03 and *p* = 0.04, respectively. Additionally, to make reporting consistent with the rest of the studies, all means have been removed from the text and reported in **Table 2**.

A correction has been made to **Experiment 2**, **Results** section, **paragraph two**:

“For each fate belief, we conducted a regression analysis to test whether that particular belief was different in the *choice* condition and the *neutral* condition compared to the *no choice* condition, which was treated as the reference group. Fatalism beliefs did not differ between the neutral and no-choice conditions [*B* = 0.15, *SE* = 0.14, *t*(194) = 1.07, *p* = 0.29], or between the choice and no-choice conditions [*B* = 0.04, *SE* = 0.13, *t*(194) = 0.29, *p* = 0.77]. Similarly, personal control beliefs did not differ between the neutral and no-choice conditions [*B* = −0.19, *SE* = 0.12, *t*(194) = −1.63, *p* = 0.10], or between the choice and no-choice conditions [*B* = −0.17, *SE* = 0.10, *t*(194) = −1.61, *p* = 0.11]. However, the belief in negotiating with fate was stronger in the no-choice condition than in either the neutral [*B* = −0.24*, SE* = 0.11*, t*(194) = 2.18*, p* = 0.03*, d* = 0.29] or choice [*B* = −0.20*, SE* = 0.09*, t*(194) = 2.02*, p* = 0.04, *d* = 0.29) conditions (see Figure 2). This result replicates the finding from Experiment 1.”

In **Experiment 3**, there was an error in reporting the degrees of freedom and *p*-value when testing mean-level differences in agreement with the biased response scales. The degrees of freedom was incorrectly reported as 216, and the *p*-value was incorrectly reported as *p* = 0.23. The degrees of freedom should have been 214, and the *p*-value should have been *p* = 0.24; the beta, standard error, and *t*-test statistics were correctly reported.

A correction has been made to **Experiment 3**, **Results** section, **paragraph one**:

“In this study, we did not find any significant difference in mean agreement with the biased response scale across conditions [*M*_Fatalism_ = 3.64, *M*_PersonalControl_ = 3.42, *M*_NegotiatingWithFate_ = 3.55; *F*(2, 214) = 1.45*, p* = 0.24]. Yet, given that group differences in agreement were observed in the subsequent experiments, we included participants' agreement to be biased scale as a covariate (as did Rattan et al., 2012, who used a similar paradigm) to address the concern that the results may be an artifact of greater agreement to certain beliefs rather than consequences of merely being exposed to different beliefs. To maintain consistency with Experiments 4–6, we controlled for participants' agreement to the biased response scales in all analyses in this experiment as well.”

In **Experiment 4**, there was an error in reporting participants' mean agreement with the biased scale in the Fatalism condition. “*M*_Fatalism_ = 3.54” was incorrectly provided instead of “*M*_Fatalism_ = 3.53.”

A correction has been made to **Experiment 4**, **Results** section, paragraph one:

“Consistent with Rattan et al. (2012), we tested if there is a difference in the mean agreement with the biased scales differed across conditions. We found a marginal difference in participants' mean agreement with the biased scale across conditions [*M*_Fatalism_ = 3.54, *M*_PersonalControl_ = 3.67, *M*_NegotiatingWithFate_ = 3.90; *F*(2,136) = 2.89, *p* = 0.06]. Thus, we included participants' agreement to be biased scale as a covariate (as did Rattan et al., 2012, who used a similar paradigm).”

Additionally, in reporting the *t*-test statistic when comparing the effects of activating fatalism vs. personal control on viewing one's own behaviors as contributing to the even [incorrectly reported as *t*(90) = 1.31, which should have been *t*(90) = 0.24]; the beta, standard error, and *p*-value were correctly reported.

A correction has been made to **Experiment 4**, **Results** section**, paragraph three**:

“The results supported our hypotheses (see Figure 4): activating negotiating with fate led participants to more readily identify behaviors as a contributing factor, compared to activating fatalism [*B* = −0.45, *SE* = 0.23; *t*(135) = 1.91, *p* = 0.06, *d* = 0.40], and activating personal control [*B* = −0.52, *SE* = 0.23; *t*(135) = 2.26, *p* = 0.03, *d* = 0.47]. Using the same procedure outlined for testing the effects of personal control vs. fatalism in Experiment 3, we conducted an additional regression. The findings indicated that activating fatalism did not lead participants to view behaviors as a contributing factor to a lesser extent than activating personal control [*B* = −0.05, *SE* = 0.22; *t*(90) = 0.24, *p* = 0.81, *d* = 0.10].”

In **Experiment 5**, there was an error in reporting participants' mean agreement with the biased scale in the negotiable fate condition. “*M*_*NegotiateWithFate*_ = 3.10” should be “*M*_*NegotiateWithFate*_ = 3.09.”

A correction has been made to **Experiment 5**, **Results** section, paragraph one:

“There was a significant difference in mean agreement with the biased scale across conditions [*M*_Fatalism_ = 3.58, *M*_PersonalControl_ = 3.40, *M*_*NegotiateWithFate*_ = 3.09; *F*(2,191) = 6.10, *p* = 0.003]. Therefore, we controlled for mean levels of agreement with the biased scale items used in the manipulation in the following analyses.”

Furthermore, the mean agreement with the biased scale across all conditions was in correctly provided as in reporting the *t*-test statistic when comparing the effects of activating negotiating with fate vs. personal control on positively reappraising the event [incorrectly reported as *t*(190) = 2.26, which should have been *t*(190) = −1.64]; the beta, standard error, and *p*-value were reported correctly for these results. There was also an error in reporting the *p*-value when comparing the effects of activating negotiating with fate and fatalism (incorrectly reported as *p* = 0.06, which should have been *p* = 0.07).

A correction has been made to **Experiment 5**, **Results** section, **paragraph three**:

“With regards to adaptive coping, participants reported significantly greater *positive reappraisal* of the event after negotiating with fate was activated compared to activating fatalism [*B* = −0.31, *SE* = 0.14, *t*(190) = 2.26, *p* = 0.03, *d* = 0.39], and marginally greater positive reappraisal when compared to activating personal control [*B* = −0.22, *SE* = 0.14, *t*(190) = −1.64, *p* = 0.10, *d* = 0.28]. For *acceptance*, participants reported marginally greater acceptance of the event after negotiating with fate was activated compared to activating fatalism [*B* = −0.22, *SE* = 0.11, *t*(190) = −1.81, *p* = 0.07, d = 0.10]. No differences in acceptance were found between the activating negotiating with fate and personal control [*B* = −0.02, *SE* = 0.11, *t*(190) = −0.20, *p* = 0.84).”

Additionally, in **Experiment 6**, there was an error in reporting the *p*-value for the differences in how helpful participants found the silver lining across experimental groups (incorrectly reported as *p* = 0.32, which should have been *p* = 0.42); the *F*-ratio was correctly reported.

A correction has been made to the **Experiment 6**, **Methods** section, subsection **Measures and Procedures**, **paragraph one**:

“Using the same instructions as previous experiments, participants were asked to recall an event in their past where they had no choice but to accept the situation. Participants were then randomly exposed to one of the biased questionnaires from Experiments 3–5 (α _Fatalism_ = 0.79; α _PersonalControl_ = 0.83; α_NegotiatingWithFate_ = 0.92). Next, participants were further asked to reflect and think of a silver lining (i.e., something positive) that they can derive from the event. Participants also answered the following question, “Did thinking of a silver lining help you to see the negative event in a more positive light?” on a 9-point scale (1 = *it made it extremely difficult* to 9 = *it made it extremely easy*). This item was used as a covariate because we wanted to investigate whether the simple *act* of asking participants to search for a silver lining was sufficient to eliminate the beneficial effects of negotiating with fate. From a theoretical perspective, we wanted to test whether the mere completion of this activity was sufficient to mimic the effects of negotiating with fate, regardless of how helpful the participant found this exercise to be. From a statistical perspective, participants' helpfulness ratings correlated significantly with meaning (*r* = 0.33, *p* < 0.001), and thus, we needed to control for individual differences in the perceived helpfulness of the task. There were no significant differences in ratings of helpfulness across groups, *F*(3,154) = 0.944, *p* = 0.42.”

In the original article, there was a mistake in Table 1 as published. We now included an extra row to state that one person in Study 5 did not report their religious affiliation to ensure that the sample size reported in the text matched the table. The corrected Table 1 appears below.

**Table 1:** Demographic information for all six samples.

	**Study 1**	**Study 2**	**Study 3**	**Study 4**	**Study 5**	**Study 6**
**Age**						
Mean	35.9	30.2	33.6	33.5	29.3	35.0
Standard Deviation	11.9	8.9	10.8	11.8	8.7	12.9
Range	19–66	18–65	19–67	18–71	18–61	18–71
**Gender**						
Men	62	111	97	48	127	72
Women	59	89	120	90	67	86
**Educational attainment**						
Did not complete high school	1	1	1	2	1	2
Completed high school	18	18	26	14	18	14
Incomplete college degree	26	54	56	42	59	52
Associate's degree	14	27	27	18	28	12
Bachelor's degree	46	72	81	46	73	59
Master's degree	12	21	22	15	11	16
Doctoral degree	4	5	4	2	3	2
Unreported	0	2	0	0	1	1
**Religious affiliation (*****n*****)**						
Non-religious	56	114	130	68	117	75
Protestant	25	27	21	22	25	27
Catholic	16	23	32	21	27	23
Christian	13	21	22	17	18	21
Other religions	11	15	12	10	6	12
Unreported	0	0	0	0	1	0

*Frequencies are reported for gender, educational attainment and religious affiliation*.

There was also a mistake in Table 2 as published. The mean value for the Negotiating Fate in Study 1 should be “4.41” instead of “4.42”. The corrected Table 2 appears below.

**Table 2:** Means, standard deviations and 95% confidence intervals for the three lay beliefs about personal control and fate in Experiments 1 and 2.

	**Choice**				**Neutral**				**No Choice**			
	**Mean**	**S.D**.	**95% Confidence interval**		**Mean**	**S.D**.	**95% Confidence interval**		**Mean**	**S.D**	**95% Confidence interval**	
			**Lower**	**Upper**			**Lower**	**Upper**			**Lower**	**Upper**
**Study 1**												
Personal control	4.57	0.62	4.36	4.77	4.46	0.73	4.25	4.66	4.40	0.57	4.20	4.61
Fatalism	2.97	0.83	2.70	3.24	3.26	0.89	3.01	3.52	3.43	0.69	3.18	3.67
Negotiating with fate	4.47	0.61	4.28	4.67	4.41	0.64	4.23	4.60	4.72	0.45	4.56	4.88
**Study 2**												
Personal control	4.00	0.55	3.86	4.13	4.00	0.58	3.86	4.14	4.14	0.54	4.01	4.28
Fatalism	2.89	0.67	2.72	3.06	2.98	0.64	2.83	3.14	2.88	0.75	2.69	3.06
Negotiating with fate	4.90	0.54	4.77	5.03	4.90	0.53	4.77	5.03	5.05	0.55	4.92	5.19

There is also a mistake in Table 4. The mean value for identifying own behavior as a contributing factor in Study 4 was incorrectly provided as “3.32” instead of “3.33”. Additionally, the upper 95% Confidence Interval of Positive reappraisal for Study 5 was incorrectly provided as “2.78” instead of “2.98”. The corrected Table 4 appears below.

**Table 4:** Means, standard deviations and 95% confidence intervals for Experiments 3–6.

	**Personal Control**				**Fatalism**				**Negotiating with Fate**			
	**Mean**	**S.D**.	**95% Confidence interval**		**Mean**	**S.D**.	**95% Confidence interval**		**Mean**	**S.D**	**95% Confidence interval**	
			**Lower**	**Upper**			**Lower**	**Upper**			**Lower**	**Upper**
**Study 3**												
Rumination	1.89	0.59	1.75	2.02	2.02	0.61	1.88	2.17	1.80	0.56	1.67	1.93
**Study 4**												
Identifying own behavior as a contributing factor	3.33	1.12	3.03	3.68	3.43	0.97	3.14	3.72	3.87	1.17	3.52	4.22
**Study 5**												
Positive reappraisal	2.63	0.78	2.45	2.84	2.57	0.76	2.38	2.76	2.80	0.76	2.60	2.98
Acceptance	3.37	0.63	3.21	3.53	3.22	0.66	3.05	3.38	3.30	0.69	3.11	3.46
Denial	1.43	0.56	1.29	1.57	1.30	0.43	1.19	1.40	1.38	0.54	1.24	1.51
Behavioral disengagement	2.05	0.65	1.89	2.22	2.02	0.59	1.88	2.17	1.98	0.62	1.83	2.14
**Study 6**												
Meaning	4.09	1.42	3.71	4.47	4.04	1.48	3.61	4.46	4.55	1.60	4.10	4.99

The authors apologize for these errors and state that these corrections do not change the scientific conclusions of the article in any way. The original article has been updated.
