# Supplementary material for: Are There Advantages to Believing in Fate? The Belief in Negotiating With Fate When Faced With Constraints
Source: Front Psychol. 2019 Nov 8;10:2354. doi: 10.3389/fpsyg.2019.02354 (PMC6857186; doi:10.3389/fpsyg.2019.02354)
Supplement: Supplementary file 1 [file Data_Sheet_1.docx]

**Appendix A: Negotiating with Fate Measure (Experiment 1)**

1. Sometimes, fate presents unexpected challenges but I can still work with it by doing the best I can.
2. There’s no situation bestowed by fate that I cannot improve upon.
3. When fate puts obstacles in front of me, it just means I need to work harder.
4. Fate gives me opportunities, but I am the one who turns those opportunities into accomplishments.
5. Putting in the effort can lead to improvements in my fate.
6. Through my actions, I can negotiate with fate for better outcomes.
7. My fate can be changed for the better if I work hard at everything I do.
8. Even though fate has not given me the easiest road, I know that I can still do great things.
9. When fate makes my life tough, I rise up to the challenge by finding ways to make the best of things.
10. I cannot change what fate has given me, but I can still achieve my dreams if I put in the effort.

*Note:* Response Scale 1 = “Strongly Disagree” to 7 = “Strongly Agree”

**Appendix B: Non-Domain Specific Lay Beliefs about Fate Measure**

**(Experiment 2)**

1. When an individual experiences difficult obstacles when pursuing a goal, he/she should:
   1. Seek ways to remove these obstacles.
   2. Seek ways to work with these obstacles to attain their goals.
   3. Accept these obstacles and consider an alternative goal.
2. When individuals look back on their lives, they:
   1. Should feel that they had control over all the important aspects of their lives.
   2. Should feel that they made the best out of the circumstances that they encountered.
   3. Should realize that their outcomes were largely determined by external forces.
3. The key to attaining important goals is:
   1. To believe that you have a lot control over the circumstances.
   2. To believe that you can work with the circumstances to attain your goals.
   3. To know when to give up.
4. In life, it is important to:
   1. Get what you want at any cost.
   2. Work with what you have to get the best that you can get.
   3. Realize that there is not much that your actions can change.
5. When individuals face a setback when pursuing a goal, they should think that:
   1. There is absolutely nothing that they cannot achieve.
   2. That they can still attain their goals if they work with the current constraints.
   3. That there is little that they can do to achieve their goals.
6. When something negative occurs, people should:
   1. Think back to all things they should have done differently.
   2. Accept the outcome and try to make the best out of a bad situation.
   3. Consider the outcome was inevitable and not affected by their actions.
7. When dealing with particularly difficult circumstances in life, people should know that:
   1. Their actions can completely change these circumstances.
   2. They can work with these circumstances to improve their current situation.
   3. There is very little that can be done to change these circumstances; they just have to accept them.

*Note:* Response scale 1 = “Strongly Disagree” to 7 = “Strongly Agree”

**Appendix C: Biased Questionnaires (Experiments 3-6)**

**Fatalism (adapted from Levenson, 1981)**

1. People’s lives are often shaped by accidental happenings.
2. Sometimes, people are unable to protect their personal interests from bad luck.
3. There are times when luck plays a role in helping people get what they want.
4. Occasionally, there are times when whatever is going to happen will happen – whether good or bad.
5. Even when people do everything right, their cars can still get hit by other drivers.
6. Often times, it might not be wise to plan too far ahead in life because many things might change by the time you get there.
7. Being at the right place at the right time can potentially affect whether a person becomes a leader.
8. Making friends requires a combination of factors that fall together—you, the other person, and the circumstances.

**Personal Control (adapted from Levenson, 1981)**

1. Most of the time, whether people get to be leaders depends on their abilities.
2. In many cases, car accidents happen because the people involved were not paying attention to their surroundings.
3. If they really wanted to, people can make plans and make them work.
4. It is usually possible for people to protect their personal interests.
5. When people get what they want, it’s to a significant extent because they worked hard for it.
6. For the most part, people’s lives are determined by their own actions.
7. In general, those who are nice to others tend to have more friends.
8. Most people are able to determine what happens in their lives.

**Negotiating with Fate**

1. By putting in the effort, it is often possible to overcome unexpected challenges that life presents us.
2. Most people can improve on their fate through their actions.
3. Often when life puts obstacles in front of people. people can improve their circumstances by working harder.
4. Although fate influences the opportunities that people get, it is often up to each person to turn those opportunities into accomplishments.
5. If people really wanted to, putting in the effort can lead to improvements in their fate.
6. For the most part, people’s fate can be changed if they work hard at everything they do.
7. Even when fate doesn’t give someone a smooth road in life, it is possible to overcome these challenges and succeed.
8. As long as people focus on making the best out of a bad situation, it doesn’t really matter what fate throws at you.

*Note:* Response scale: 1=“Disagree”, 2=“Agree Somewhat”, 3=“Agree Moderately”, 4=“Agree Quite a Bit”, 5=“Agree Strongly”
